# Quality of life of Nigerians living with human immunodeficiency virus

**DOI:** 10.11604/pamj.2014.18.234.2816

**Published:** 2014-07-22

**Authors:** Adeolu Oladayo Akinboro, Suliat Omolola Akinyemi, Peter B Olaitan, Ajani Adeniyi Raji, Adetoun Adetayo Popoola, Opeyemi Roseline Awoyemi, Olugbenga Edward Ayodele

**Affiliations:** 1 Department of Internal Medicine, Ladoke Akintola University of Technology, Osogbo, Osun State, Nigeria; 2 People Living with HIV/AIDS Clinic, Ladoke Akintola University of Technology Teaching Hospital, Osogbo, Osun State, Nigeria; 3 Department of Surgery, Ladoke Akintola University of Technology, Osogbo; 4 Department of Haematology, Ladoke Akintola University of Technology, Osogbo; 5 Department of Nursing, Ladoke Akintola University of Technology Teaching Hospital, Osogbo

**Keywords:** Quality of life (QOL), Human immunodeficiency virus, acquired immune deficiency syndrome (AIDS), people living with HIV/AIDS, Nigeria

## Abstract

**Introduction:**

Few reports from Nigeria have examined the quality of life (QOL) of people living with HIV/AIDS (PLWHA) despite the fact that Nigeria has the second largest number of PLWHA in the world. This study evaluated the QOL of Nigerians living with HIV/AIDS using the World Health Organization Quality of Life Questionnaire for HIV-Brief Version (WHOQOL-BREF) instrument and assessed the impact of demographic, laboratory and disease-related variables on QOL.

**Methods:**

This cross-sectional study involved 491 consecutive PLWHA aged ≥ 18 years attending the dedicated clinic to PLWHA in South-west Nigeria.

**Results:**

The lowest mean QOL scores were recorded in the environment and social domains. Participants aged ≥ 40 years had better QOL in the environment (p = 0.039) and spirituality (p = 0.033) domains and those in relationships had better QOL in the social relationship domain (p = 0.002). Subjects with no or primary education and those who rated their health status as good gave significantly higher ratings in all QOL domains. Participants with AIDS had significant lower QOL in the level of independence domain (p = 0.018) and those with CD4 count ≥ 350 cells /mm3 had better QOL scores in the physical, psychological and level of independence domains. Subjects without tuberculosis co-infection and those on antiretroviral therapy (ART) reported significantly better QOL in the physical, psychological, level of independence and spirituality domains.

**Conclusion:**

Marital relationship, absence of tuberculosis, CD4 count ≥ 350 cells /mm3 and use of ART positively impacted QOL of our patients.

## Introduction

Sub-Saharan Africa (SSA) bears an inordinate burden of human immunodeficiency virus (HIV) as shown by the number of people living with HIV/AIDS (PLWHA) and deaths arising from acquired immune deficiency syndrome (AIDS)-related causes [1]. An estimated 23.3 million, representing 69% of the global 34 million PLWHA reside in SSA. Also, the estimated 1.2 million adults and children who died of HIV-related illnesses in SSA in 2011 represented 70% of global 1.8 million deaths attributable to the epidemic [1]. In addition, HIV/AIDS was responsible for 46,653,000 disability-adjusted life years (DALYs) in Africa which represented approximately 80% of the global DALYs from HIV/AIDS in 2004 [2]. The prevalence of HIV/AIDS in Nigeria increased from 1.8% in 1991 to 5.8% in 2001 with a gradual fall to 4.1% in 2012 [3]. Nigeria with an estimated population of PLWHA of approximately 3.5 million has the second highest number of PLWHA in the world after South Africa [3].

The advent of highly active antiretroviral therapy (HAART) has been associated with improved clinical and laboratory outcomes, which in turn has translated to fewer opportunistic infections and overall reductions in morbidity and mortality [4–6]. However, the need for life-long medication therapy, medication side effects, and the constant stigma, discrimination and prejudice experienced by PLWHA have raised concern about other domains of health such as overall physical and mental health functioning and socioeconomic and spiritual wellbeing [7]. These domains of health which serve as indicators of quality of life (QOL) have emerged as important factors in HIV/AIDS management [7].

Quality of life is a multidimensional and subjective concept and currently there is no consensus definition of QOL [4–6]. The World Health Organization Quality of Life (WHOQOL) group defines quality of life as individuals’ perceptions of their position in life in the context of culture and value systems in which they live, and in relation to their goals, expectations, standards and concerns [8, 9]. The term health-related quality of life (HRQOL) is often used to indicate QOL as it relates to diseases or treatments [10].

The measure of QOL is of both public health and clinical research significance since it showcases the definition of health according to World Health Organization (WHO) as a “complete state of physical, mental and social wellbeing - not merely the absence of disease or infirmity” [8, 9]. Studies on QOL provides an estimation of the impact of treatment in chronic diseases for which improvement in functional status and wellbeing can be regarded as an essential outcome [10, 11]. An improved QOL and resultant ability of the patient to resume normal life, including supporting the families and working productively will encourage long-term sustenance of treatment [7]. However, if QOL is poor, it impacts negatively on life-long adherence to medication [7]. Quality of life also serves as an indicator of prognosis among patients with HIV as those from lower quartiles of physical and mental scores have a higher incidence of mortality [11].

The methodologies for assessing QOL and socioeconomic wellbeing vary greatly in published literature since different HRQOL instruments with established validity and reliability were employed [4–6, 12–24]. Studies have shown that the HRQOL in PLWHA is lower than those without HIV/AIDS and HRQOL improves with HAART [4–6, 12–16]. While some studies only assessed the HRQOL in PLWHA, other studies further assessed the clinical predictors of QOL [4–6, 12–24]. Since HRQOL has to do with individuals’ perceptions of their position in life in the context of culture and value systems in which they live, the clinical predictors of HRQOL vary in different studies and this makes it difficult extrapolating the findings in one centre or country to the other.

Most of the publications on QOL in sub-Saharan Africa had come from South Africa, Zimbabwe, and Uganda [15–17, 19–22]. There is a dearth of literature or publications on HRQOL in PLWHA from Nigeria despite the fact that Nigeria has the second highest number of PLWHA in the world [20–22]. Also, only few published studies from Nigeria [21]. have used the World Health Organization Quality of Life Brief Version (WHOQOL-BREF) instrument which is disease-specific and has a proven cross-cultural validity [8, 9]. In addition, the numbers of participants involved in these published studies were rather small [20–22]. This study evaluated the QOL of Nigerians living with HIV/AIDS using the WHOQOL-BREF instrument and assessed the impact of demographic, laboratory and disease-related variables on QOL.

## Methods

This was a cross-sectional study carried out at the dedicated Outpatient Clinic to PLWHA of Ladoke Akintola University of Technology Teaching Hospital (LTH), Osogbo, Osun State, Nigeria. The clinic is being jointly run by the Institute of Human Virology under sponsorship by the President's Emergency plan for AIDS Relief (PEPFAR) and LTH Management. The inclusion criteria for the study were PLWHA aged ≥ 18 years who gave informed oral and written consent to be part of the study. Exclusion criteria were acute illness that required medical or surgical treatment or admission; pregnancy; gross cognitive dysfunction and refusal to be part of the study.

The sample size was calculated using the Cochran's sample size formula for categorical data i.e. n = (t)2(p)(q) / d2 where t is the value for selected alpha level of 0.025 in each tail = 1.96; d is the acceptable margin of error = 5%; and (p)(q) is the estimate of variance = 0.25.25 Although the estimated sample size was 385, we recruited 504 participants over a six months period (July 2010 and Jan. 2011). A non-probabilistic consecutive sampling method was used. Only 491 (97.4%) participants had analyzable data.

The instrument used was the WHOQOL-HIV BREF [8, 9]. which is a multidimensional, conceptualized, generic, 31-item QOL instrument [8, 9]. The questions in this instrument cover the respondent's perception of the overall quality of life within the following 6 broad domains (facets) of the quality of life that summarize that particular domain: [8, 9]. (1)Physical health domain has 4 facets: pain and discomfort, energy and fatigue, sleep and rest and symptoms related to HIV; (2)Psychological health domain has 5 facets: positive feelings, concentration, self-esteem, bodily image and appearance and negative feelings; (3)Level of independence domain has 4 facets: mobility, activities of daily living, dependence on medication and treatment and work capacity; (4)Social relationship domain has 4 facets: personal relationships, social support, sexual activity, and social inclusion, (5)Environmental domain has 8 facets: physical safety and security, home environment, financial resources, health and social care, accessibility and quality, opportunities for acquiring new information and skills, participation in and opportunities for recreation/leisure activities, physical environment (pollution/noise/traffic/climate), and transport; (6)Spirituality, religion and personal beliefs domain has 4 facets: personal beliefs, forgiveness and blame, concerns about the future, death and dying.

There were two questions about general QOL and perceived general health. Domain scores were scaled in a positive direction with higher scores denoting higher QOL. Each item was rated on a 5 point LIKERT scale where 1 indicates low, negative perception and 5 indicates high, positive perception. Some items such as pain and discomfort, dependence on medication, death and dying, and negative feelings (Q3, Q4, Q5, Q8, Q9, Q10, and Q31) were not scaled in a positive direction, meaning that for these facets higher scores do not denote higher quality of life. To transform these scores in a positive direction, the formula: 6 - x (where x was the facet score) was used. The mean score of items within each domain was used to calculate the domain score.

Patients who were literate were given the questionnaire to complete themselves after going through the questionnaire with them. In the case of patients who were not literate or who were not comfortable with English Language, a “Yoruba” version of the instrument was administered face-to-face by a trained Treatment Support Specialist or a Specialist Nurse in a private room. The Yoruba version of the instrument was produced using two translators/linguists who were fluent in both languages. The first translator translated the English version of the questionnaire to “Yoruba” language. The translated “Yoruba” version was then given to the second linguist to back-translate to English. Translators were encouraged to report contentious areas and difficulties that were encountered. Thereafter, the two translators reconciled any difficult section. The authors then compared the “forward-translated” and the “back-translated versions” to ensure that there were no contentious and/or ambiguous section of the questionnaire.

The weight (kilogram) of each participant was taken in light clothing with the shoes off and the height (meters) was done using a stadiometer. Body mass index (BMI) was calculated from the formula: weight (kg) / (height)2 (m)2. Overweight and obesity were defined as BMI of 25-29.9 kg/m2 and ≥30 kg/m2 respectively [26]. Patients’ WHO stage, CD4 count, previous body weights before commencement of study, height, packed cell volume and serum creatinine were obtained from the case notes.

Ethical approval for the study was obtained from the Research Ethics Committee of our institution (LTH) Osogbo, Nigeria.

### Statistical Analysis

The mean scores of items within each domain of the WHOQOL-HIV BREF were used to calculate the domain score. Mean scores were then multiplied by 4 in order to make domain scores comparable with the scores in the full version of the World Health Organization Quality of Life instrument (WHOQOL-100). Continuous and categorical variables were displayed as means ± standard deviation (S.D) and percentages respectively. Variables such as age, marital status, BMI, CD4 count, PCV, and treatment duration were dichotomised. The duration of treatment was dichotomized using the median duration of treatment as cut-off. The student's t test was used to assess differences between means of individual variables and differences in the mean scores of various domains of the WHOQOL-HIV BREF. Differences between categorical variables were analyzed by Chi-square test with Fisher's exact correction applied as appropriate. Differences between ≥3 groups were analyzed using analysis of variance (ANOVA). All p values were two-tailed and values <0.05 were considered to be statistically significant. All statistical analyses were done using Statistical Package for Social Sciences (SPSS) software, version 15 (SPSS, Chicago, IL, USA).

## Results

The socio-demographic and clinical characteristics of the study population are presented in Table 1. The study population consisted of 491 patients (144 (29.3%) males and 347 (70.7%) females). The women were significantly younger than the men with 67.7% of the women being <40 years. Majority of the participants (65.4%) had high school and tertiary education and 247 (50.7%) were married as at the time of data collection, with the men more likely to be married (63.9% vs. 45.2%). On self-evaluation of health status 202 (41.7%), 228 (47.1%), 40 (8.3%), 8 (1.7%) and 6 (1.2%) rated their health as very good, good, neither good nor bad, poor and very poor respectively. The proportion of our patients with AIDS was significantly higher than those with asymptomatic and symptomatic disease (p = 0.005). Two hundred and forty seven participants (53.5%) could not ascertain the source of infection while 121 (26.2%) admitted to sexual relationship as the source of infection. Fifteen (3.1%) patients had tuberculosis at the time of data collection. A total of 393 (80%) patients were on HAART and the mean duration of treatment was 18.21 months (median 18.00 months, range 1 - 63 months).

**Table 1:** Socio-demographic and clinical characteristics of the study population

Characteristics	Male (%)	Female (%)	Total (%)	P value
Gender	144 (29.3)	347 (70.7)	491 (100)	<0.001
Mean age±SD (yrs)	43.3±10.4	36.6±8.7	38.5±9.7	<0.001
**Educational status**				**0.820**
None	14 (9.7)	38 (11.0)	52 (10.6)	
Primary	33 (22.9)	85 (24.5)	118 (24.0)	
Secondary	52 (36.1)	130 (37.5)	182 (37.1)	
Tertiary	45 (31.3)	94 (27.1)	139 (28.3)	
**Marital status**				**<0.001**
Single	22 (15.3)	42 (12.2)	64 (13.1)	
Married	92 (63.9)	155 (45.2)	247 (50.7)	
Living as married	10 (6.9)	35 (10.2)	45 (9.2)	
Separated	9 (6.3)	30 (8.7)	39 (8.0)	
Divorced	1 (0.7)	17 (5.0)	18 (3.7)	
Widowed	10 (6.9)	64 (18.7)	74 (15.2)	
**Self-evaluated health status**				**0.118**
Very poor	2 (1.4)	4 (1.2)	6 (1.2)	
Poor	5 (3.5)	3 (0.9)	8 (1.7)	
Neither poor nor good	10 (7.0)	30 (8.8)	40 (8.3)	
Good	74 (52.1)	154 (45.0)	228 (47.1)	
Very good	51 (35.9)	151 (44.2)	202 (41.7)	
**Perceives having an illness**				**0.296**
Yes	38 (26.4)	108 (31.1)	146 (29.7)	
No	106 (73.6)	239 (68.9)	345 (70.3)	
**HIV serostatus**				**0.005**
Asymptomatic	20 (13.9)	77 (22.2)	97 (19.8)	
Symptomatic	31 (21.5)	101 (29.1)	132 (26.9)	
AIDS	93 (64.6)	169 (48.7)	262 (53.3)	
**Source of infection (n =462)**				**0.986**
Sex	34 (25.8)	87 (26.4)	121 (26.2)	
Injection	8 (6.1)	18 (5.5)	26 (5.6)	
Blood products	14 (10.6)	30 (9.1)	44 (9.5)	
Sharp	7 (5.3)	17 (5.2)	24 (5.2)	
Don't know	69 (52.3)	178 (53.9)	247 (53.5)	
**Associated Tuberculosis**				**0.134**
Yes	7 (4.9)	8 (2.3)	15 (3.1)	
No	137 (95.1)	339 (97.7)	476 (96.9)	
**On HAART**				**0.575**
Yes	113 (78.5)	280 (80.7)	393 (80.0)	
No	31 (21.5)	67 (19.3)	98 (20.0)	
Mean weight±SD (kg)	68.0±11.0	62.1±13.2	63.8±12.9	<0.001
Mean height±SD (m)	1.70±0.07	1.61±0.07	1.63±0.08	<0.001
Mean BMI±SD (kg/m^2^)	23.6±3.8	24.1±4.9	23.9±4.58	0.329
**BMI group (kg/m** ^**2**^ **) (n = 485)**				**0.094**
<18.5	9 (6.3)	33 (9.6)	42 (8.7)	
18.5 – 24.9	86 (60.1)	189 (55.3)	275 (56.7)	
25.0 – 29.9	40 (28.0)	80 (23.4)	120 (24.7)	
≥30	8 (5.6)	40 (11.7)	48 (9.1)	
Mean Serum Cr±SD (µmol/L)	101.2 ± 24.9	83.1 ± 34.7	88.5±33.1	<0.001
Mean PCV±SD (%)	37.7±13.6	32.8 ± 4.3	34.2±8.5	0.001
Mean CD4±SD (cells/mm^3^)	287.0±213.0	362.0±234.0	349.0±230.0	0.001

Key: HIV – Human immunodeficiency virus, SD – standard deviation, HAART – highly active antiretroviral therapy, BMI – body mass index, Cr – creatinine, eGFR – estimated glomerular filtration rate, PCV – packed cell volume.

The males were significantly taller than the females. Also, the mean weight of the males was significantly higher than the females though there was no statistically significant difference in the mean BMI (p = 0.329). A total of 42 (8.7%), 120 (24.7%) and 48 (9.1%) were malnourished, overweight and obese respectively. The males have significantly higher serum creatinine and packed cell volume than the females. However, the mean CD4 count was significantly higher in the females compared to the males (362 ± 234 vs. 287 ± 213.0, p = 0.001).

Table 2 presents the internal consistency validity and Spearman's correlation between each WHOQOL-BREF domain score with the two general items, the overall QOL and general health perception. The lowest mean scores were in the environment and social relationship domains whilst the highest score was in the spirituality domain. The Cronbach's alpha ranged from 0.81 to 0.85 across the six domains indicating good internal consistency reliability. The alpha value for the whole scale was 0.85. The scores for all the QOL domains positively correlated with overall quality of life questions, general health satisfaction, and self-evaluated health status (Spearman's rho range: 0.152 - 0.371, 0.104 - 0.390 and 0.144 - 0.319 across domains respectively) (Table 2).

**Table 2:** Mean scores, internal consistency reliability and Spearman's rank correlation between WHOQOL-BREF domains scores and health status measures

Domain	Mean (SD) scores	Cronbach's alpha	Overall quality of life	General health perceptions	Self-evaluated health status
Physical	16.85±2.79	0.81	0.294†	0.285†	0.272†
Psychological	16.23±2.60	0.81	0.371†	0.390†	0.302†
Level of independence	16.33±2.73	0.82	0.268†	0.318†	0.319†
Social relationship	16.09±2.81	0.83	0.237†	0.197†	0.166†
Environment	16.08±2.54	0.83	0.262†	0.127†	0.124*
Spirituality	16.93±3.28	0.85	0.152†	0.104*	0.104†

* Note: p<0.05

† p<0.001

The inter-domain correlations showed significant association between all the domains of the entire test instrument (Table 3). Strong correlations were observed between level of independence and physical domains (r = 0.677, p < 0.001). The weakest correlation was observed between spirituality and social relationship domains (r = 0.348, p < 0.001).

**Table 3:** Correlation matrix between the domains of the WHOQOL-BREF Questionnaire

	Physical	Psychological	Level of independence	Social relationship	Environment	Spirituality
Physical	1.000					
Psychological	0.595*	1.000				
Level of independence	0.677*	0.606*	1.000			
Social relationship	0.422*	0.507*	0.441*	1.000		
Environment	0.418*	0.549*	0.445*	0.628*	1.000	
Spirituality	0.518*	0.434*	0.415*	0.348*	0.377*	1.000

* Note: p<0.001

Comparison of the mean scores of quality of life according to socio-demographic, clinical and disease-related characteristics is presented in Table 4. There was no statistically significant gender difference in the mean scores in all the QOL domains. Participants who were ≥40 years had significantly higher mean QOL scores in the environment and spirituality domains when compared to those who were <40 years. Participants who were married had significant higher mean score in the social relationship domain when compared to others. Participants with no education and primary education had statistically significant higher mean scores in all the QOL domains with the exception of social relationship domain though this was not statistically significant. Patients who rated their health status as being very good/good reported significantly better QOL in all the domains when compared with those who rated their health status as very poor/poor and neither poor nor good. Participants who perceived themselves as being ill had lower mean QOL scores in all the domains. Patients with AIDS had lower mean scores in all the QOL domains except spirituality domain when compared with those with asymptomatic and symptomatic disease although this was only statistically significant for the level of independence domain.

**Table 4:** Comparison of mean scores of quality of life according to socio-demographic, clinical and disease-related characteristics

Variables	Physical	Psychological	Level of independence	Social relationship	Environment	Spirituality
**Gender**						
Male (n = 144)	16.72±3.06	16.21±2.66	16.03±2.96	16.13±2.76	16.16±2.47	16.78±3.33
Female (n = 347)	16.90±2.58	16.25±2.58	16.45±2.62	16.07±2.83	16.05±2.58	16.99±3.27
P value	0.510	0.873	0.128	0.830	0.665	0.518
**Age group (years)**						
< 40 (n = 287)	16.83±2.86	16.11±2.66	16.34±2.69	15.90±2.96	15.89±2.72	16.67±3.42
≥ 40 (n = 204)	16.88±2.71	16.40±2.51	16.30±2.79	16.36±2.55	16.36±2.25	17.29±3.06
P value	0.861	0.224	0.854	0.069	0.039	0.033
**Educational status**						
None (n = 52)	17.08±2.50	16.85±2.50	17.12±2.10	16.77±2.18	16.69±2.25	17.33±3.35
Primary (n = 118)	17.34±2.50	16.81±2.42	16.52±2.65	16.40±2.38	16.61±2.25	17.36±2.87
Secondary (n = 182)	16.90±2.66	16.05±2.48	16.45±2.62	15.92±2.97	15.87±2.68	17.06±3.18
Tertiary (n = 139)	16.30±3.20	15.76±2.82	15.71±3.03	15.79±3.07	15.69±2.61	16.24±3.62
P value	0.023	0.002	0.006	0.082	0.005	0.026
**Marital status**						
Married/living as married (n = 292)	16.99±2.76	16.31±2.51	16.43±2.72	16.41±2.66	16.20±2.52	16.96±3.24
Others (n = 195)	16.66±2.85	16.15±2.72	16.17±2.76	15.61±2.96	15.91±2.58	16.91±3.35
P value	0.193	0.500	0.309	0.002	0.209	0.857
**Self-evaluated health status**						
Very poor/poor (n = 14)	12.21±3.87	12.46± 3.15	12.43±2.79	14.71±4.18	15.96±3.49	14.14±4.32
Neither poor nor good (n = 40)	14.58±2.95	13.78± 2.66	13.83±2.76	14.50±3.32	14.55±2.67	15.43±3.78
Very good/good (n = 430)	17.23±2.49	16.63±2.30	16.71±2.48	16.28±2.65	16.25±2.45	17.16±3.14
P value	<0.001	<0.001	<0.001	<0.001	<0.001	<0.001
**Perceives having an illness**						
Yes (n = 146)	15.69±3.08	15.25±2.68	15.37±2.74	15.75±2.78	15.73±2.40	16.38±3.51
No (n = 35)	17.34±2.51	16.65±2.45	16.73±2.62	16.23±2.81	16.23±2.59	17.16±3.16
P value	<0.001	<0.001	<0.001	0.078	0.047	0.021
**HIV Serostatus**						
Asymptomatic (n = 97)	16.95±2.69	16.48±2.42	16.54±2.54	16.36±2.94	16.40±2.51	16.91±3.42
Symptomatic (n = 132)	17.18±2.47	16.41±2.46	16.80±2.25	16.14±2.70	16.03±2.49	16.89±3.23
AIDS (n = 262)	16.65±2.97	16.06±2.72	16.01±2.97	15.96±2.81	15.99±2.58	16.95±3.27
P value	0.188	0.268	0.018	0.474	0.394	0.985
**Body mass index (kg/m2)**						
<25 (n = 317)	16.73±2.80	16.12±2.78	16.06±2.85	15.91±2.87	15.87±2.60	16.72±3.37
≥25 (n = 168)	17.04±2.75	16.45±2.23	16.82±2.34	16.38±2.66	16.48±2.39	17.33±3.10
P value	0.244	0.164	0.002	0.083	0.012	0.053
**Associated Tuberculosis**						
Yes (n = 15)	14.60±2.56	14.93±2.78	13.93 ± 2.09	16.00 ± 2.78	16.60 ± 2.11	15.47 ± 3.83
No (n = 476)	16.92±2.77	16.28±2.60	16.40 ± 2.71	16.09 ± 2.81	16.07 ± 2.56	16.97 ± 3.26
P value	0.001	0.049	0.001	0.900	0.424	0.08
**CD4 count (cells /mm3)**						
<350 (n = 299)	16.59±2.89	16.01±2.69	16.00±2.81	16.03±2.79	16.04±2.49	16.84±3.21
≥350 (n = 192)	17.27±2.59	16.59±2.43	16.83±2.52	16.19±2. 83	16.15±2. 62	17.07±3.40
P value	0.008	0.016	0.001	0.536	0.646	0.446
**PCV (%)**						
< 30 (n = 74)	16.31±3.05	15.65±2.99	15.95±3.02	16.28±2.65	16.05±2.38	17.00±3.02
≥ 30 (n = 416)	16.95±2.74	16.35±2.51	16.39±2.67	16.06±2.83	16.10±2.57	16.93±3.30
P value	0.070	0.064	0.235	0.532	0.893	0.875
**On HAART**						
Yes (n = 393)	17.16±2.55	16.43±2.48	16.52±2.66	16.09±2.81	16.00±2.56	17.09±3.16
No (n = 98)	15.60±3.33	15.45±2.90	15.56±2.86	16.08±2.79	16.38±2.50	16.26±3.67
P value	<0.001	0.002	0.002	0.975	0.184	0.040
**Treatment duration**						
≤18 months (n = 200)	16.96±2.72	16.26±2.66	16.30±2.78	16.15±2.92	15.97±2.59	16.80±3.47
>18 months (n = 193)	17.34±2.41	16.48±2.33	16.74±2.49	16.07±2.82	16.09±2.61	17.34±2.86
P value	0.163	0.403	0.112	0.796	0.649	0.104

Key: HIV – Human immunodeficiency virus, AIDS – Acquired immune deficiency syndrome, PCV – packed cell volume, HAART – highly active antiretroviral therapy.

Participants with BMI ≥ 25 kg/m2 reported significantly better QOL for level of independence and environment domains than those with BMI < 25 kg/m2. Those with tuberculosis had statistically significant lower mean scores in the physical, psychological and level of independence domains when compared with those without TB. When compared with participants with CD4 count < 350 cells/mm3, those with CD4 count ≥ 350 cells/mm3 had higher mean scores in all the QOL domains and these were statistically significant in the physical, psychological and level of independence domains. The packed cell volume did not significantly impact on the mean QOL scores. Participants who were on HAART reported significantly better QOL in the physical, psychological, level of independence and spirituality domains when compared with those who were not on HAART. Participants who had been on HAART for more than 18 months had higher mean scores in all the domains with the exception of social domain when compared with those who had been on HAART for 18 months or less although the differences in mean scores were not statistically significant.

## Discussion

Approximately 71% of our study population were females consistent with various reports from SSA which showed the greater burden of HIV/AIDS in females [5, 7, 15, 16, 19–22]. The lowest mean QOL scores were obtained in the environment and social domains. These findings are consistent with published studies and can be explained by the fact that PLWHA often experience social isolation, stigmatization, discrimination and marginalization [17, 18, 27]. The highest QOL scores were recorded in the spirituality domain consistent with reports from Africa [21, 24]. Africans are generally religious particularly when confronted with life issues that defy medical solutions and this may explain why the highest score was obtained in the spirituality domain [21].

We did not find any significant gender difference in all the QOL domains unlike many reports which showed better QOL in males compared to females [18, 20]. However, our findings are similar to reports of Perez et *al*. [14]. and Kovacević et *al*. [17]. which did not show any gender difference in the QOL of PLWHA. Patients who were ≥ 40 years had significantly better QOL in the environment and spirituality domains. Unlike other reports which showed better QOL in younger patients, [17]. those aged <40 years did not show significant better QOL in the physical, psychological and level of independence domains.

Unlike many reports [13, 15, 17]. that documented better QOL in well educated people, we found that subjects with no formal and primary education reported better QOL in all domains. However, our finding was similar to that by Abboud et *al*. [18]. which showed better QOL in those with no education or elementary education. A possible explanation for this finding is that educated people may be more enlightened about the disease, its complications and the alterations in lifestyle needed to prevent the transmission of the disease to others may negatively impact on their QOL [18].

Consistent with many reports, [12, 17, 18]. we found a significant better QOL in the social relationship domain in subjects who were married or in relationship when compared to those who were separated, single or had lost their spouses. It is well known that the family setting provides safety, security and financial support. Thus, those who were married likely enjoyed better social support, closer interpersonal relationship and satisfactory sexual activity which in turn impact positively on the QOL [17, 18].

Participants who reported being currently ill had poorer QOL in all the domains, a finding consistent with findings by Kovacević et *al*. [17]. The preoccupation of patients with this disease and the physical symptoms they experienced can impact negatively on QOL.

Studies have not shown a consistent association between stages of HIV infection and QOL domains. While some studies have shown reduced QOL with severity of disease, [13, 27]. others did not [17]. Wig et *al*. [13]. documented significant differences in the physical and psychological domain scores in different clinical categories of HIV patients while Rai et *al*. [27]. reported that patients with AIDS experienced significantly poor QOL in all the six QOL domains when compared with those who were asymptomatic and symptomatic. On the other hand, Kovacevic et *al*. [17]. did not find any significant differences among QOL domains with stages of HIV infection. We found a significant reduction in the level of independence of participants with AIDS when compared with asymptomatic and symptomatic participants. A possible explanation of this finding is that patients with AIDS depend more on others and medication and have less capacity for work due to weakness and decline in health.

Our patients who were overweight /obese had better QOL in all the domains although this was only statistically significant in the level of independence and environment domains. The sight of a lean or wasted individual may affect acceptability and integration into the society and therefore may hinder social relationship and interactions [28]. Also, patients with weight loss may live continuously in torment of suspense that other people are aware of their serology status which may lead to depression and withdrawal from public functions. From the foregoing, societal acceptability, social relationship and interactions and psychological wellbeing may be better in PLWHA who were overweight/obese. This may explain why our patients who were overweight /obese had better QOL in all the domains.

Participants with HIV/TB had significantly lower QOL in the physical, psychological and level of independence domains when compared with PLWHA without TB. This finding is consistent with that by Deribew et *al*. [24]. However, there is no significant differences in the mean scores in the social relationship, environment and spirituality domains unlike the report by Deribew et *al*. [24]. It is believed that the occurrence of two stigmatizing diseases can impact negatively on the QOL of the patients [24]. Unlike the study by Deribew et *al*., [24]. we found a significant association between CD4 count and QOL with participants with CD4 count ≥ 350 cells/mm3 reporting better QOL in the physical, psychological and level of independence domains. Anemia has been shown to negatively impact on the physical and psychological well-being as well as social functioning in PLWHA [29]. Thus, it is not surprising that our patients with PCV ≥ 30% had higher mean QOL scores which approached significance in the physical and psychological domains.

Published reports have shown that participants on HAART reported significant improvements in physical health, emotional well-being and mental health, reduced absenteeism from work, improved work productivity and performance when compared with those not yet on treatment [7, 15, 16, 19]. In our cohort, participants who were on HAART had better QOL in the physical, psychological, level of independence and spirituality domains when compared with those who were not on HAART, consistent with published studies [15, 16, 19]. Although some workers have raised the possibility that the side effects from HAART may impact negatively on QOL, many studies in the post-HAART era have shown improvement in self-reported QOL in PLWHA [15, 16]. When compared with those who had been on HAART for 18 months or less, participants who had been on HAART for more than 18 months had higher, albeit non-significant mean scores in all the domains except the social domain. Jelsma et *al*.showed that there is progressive improvement in the QOL of South Africans living with HIV in a longitudinal study. The cross-sectional nature of our study makes it difficult to demonstrate the improvement in QOL with increasing duration on HAART which will require a longitudinal study design.

The strength of our study is that the study population is larger than most published studies from Nigeria [20–22]. and we used the WHOQOL-BREF instrument which is disease-specific and has been shown to have cross-cultural validity [8, 9].

Our study has some limitations. First, in view of the fact that the WHOQOL-BREF instrument measures QOL within two weeks prior to the interview, the information provided may be influenced by recall bias. Second, the cross-sectional design of the study makes it difficult to causally link or draw conclusions on the direction of the relationship of the socio-demographic and disease-related variables with QOL. Third, the study population consisted of consecutive patients seen in a tertiary centre and our findings may not necessarily apply to the generality of PLWHA in Nigeria.

## Conclusion

The QOL of our patients in the social and environment domains were not as good as other domains. This underscores the need to improve social support and personal relationships of our patients and to provide a supportive environment without discrimination, stigmatization and marginalization which in turn will allow our patients to thrive socially, physically and financially. Although we were not able to show significant gender difference in all the QOL domains, the brunt of HIV/AIDS is still being borne by females as shown by the preponderance of females in our study population. Marital relationship, the absence of tuberculosis, the absence of wasting, CD4 count ≥ 350 cells /mm3 and use of HAART positively impacted QOL of our patients.
